# Genetic Testing of Maturity-Onset Diabetes of the Young Current Status and Future Perspectives

**DOI:** 10.3389/fendo.2018.00253

**Published:** 2018-05-17

**Authors:** Parveena Firdous, Kamran Nissar, Sajad Ali, Bashir Ahmad Ganai, Uzma Shabir, Toyeeba Hassan, Shariq Rashid Masoodi

**Affiliations:** ^1^Centre of Research for Development (CORD), University of Kashmir, Srinagar, India; ^2^Department of Biochemistry, University of Kashmir, Srinagar, India; ^3^Department of Endocrinology, Sher-I-Kashmir Institute of Medical Sciences, Srinagar, India

**Keywords:** maturity-onset diabetes of the young, gene mutation, diabetes, hyperglycemia, sulfonylureas, insulin

## Abstract

Diabetes is a global epidemic problem growing exponentially in Asian countries posing a serious threat. Among diabetes, maturity-onset diabetes of the young (MODY) is a heterogeneous group of monogenic disorders that occurs due to β cell dysfunction. Genetic defects in the pancreatic β-cells result in the decrease of insulin production required for glucose utilization thereby lead to early-onset diabetes (often <25 years). It is generally considered as non-insulin dependent form of diabetes and comprises of 1–5% of total diabetes. Till date, 14 genes have been identified and mutation in them may lead to MODY. Different genetic testing methodologies like linkage analysis, restriction fragment length polymorphism, and DNA sequencing are used for the accurate and correct investigation of gene mutations associated with MODY. The next-generation sequencing has emerged as one of the most promising and effective tools to identify novel mutated genes related to MODY. Diagnosis of MODY is mainly relying on the sequential screening of the three marker genes like hepatocyte nuclear factor 1 alpha (HNF1α), hepatocyte nuclear factor 4 alpha (HNF4α), and glucokinase (GCK). Interestingly, MODY patients can be managed by diet alone for many years and may also require minimal doses of sulfonylureas. The primary objective of this article is to provide a review on current status of MODY, its prevalence, genetic testing/diagnosis, possible treatment, and future perspective.

## Introduction

**Figure 1:**
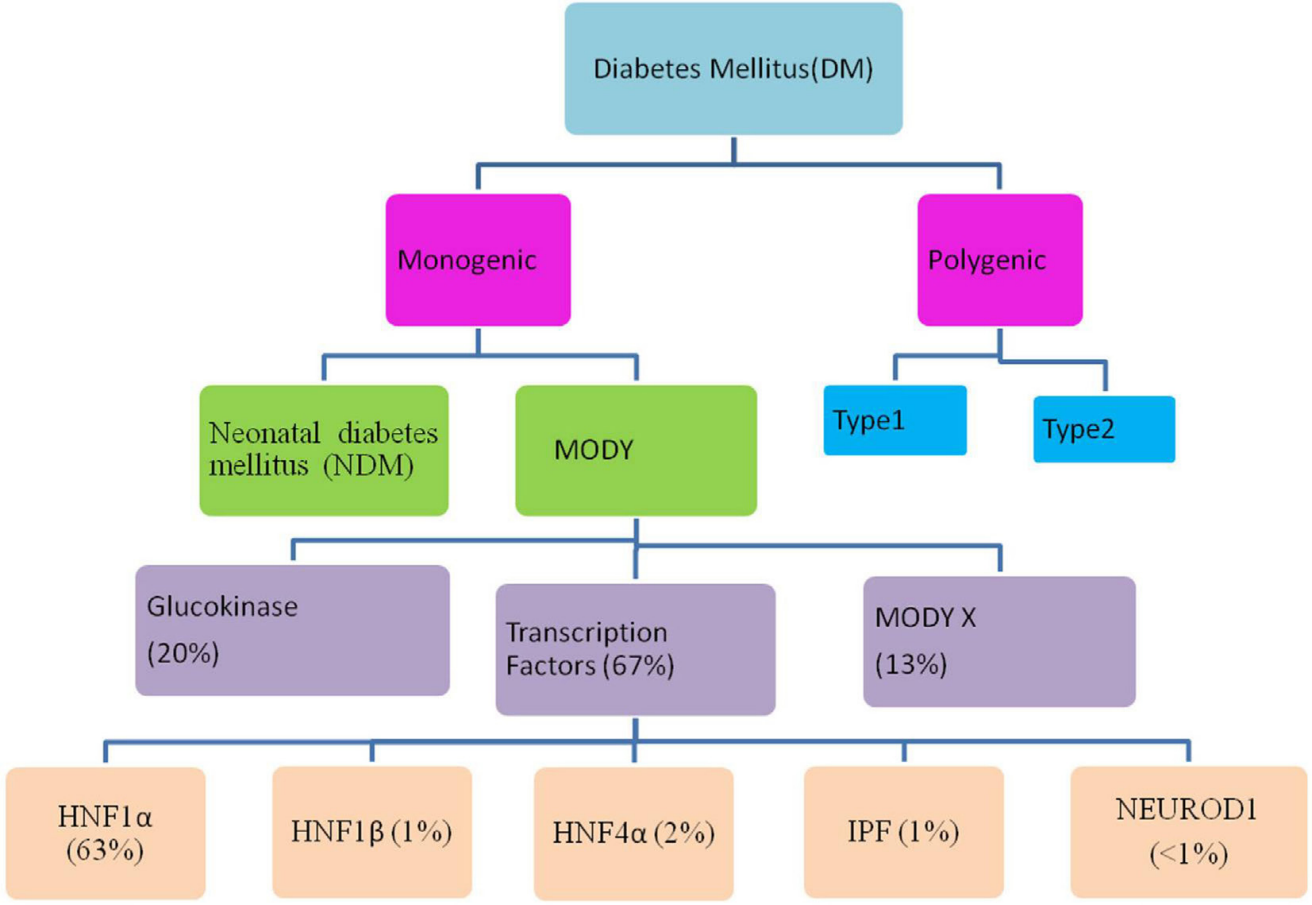
Flow through diagram showing types of diabetes mellitus including the relative prevalence of various maturity-onset diabetes of the young (MODY) causing genes.

Maturity-onset diabetes of the young (MODY) is a monogenic, clinically and genetically heterogeneous form of diabetes showing autosomal dominant mode of inheritance spanning up to three generations. It accounts for 1–5% of all the diabetic forms of young and is specified by anomalous pancreatic β-cell activity (1–3). Neonatal diabetes mellitus (NDM) and MODY are the two monogenic forms of diabetes, with MODY showing higher occurrence than NDM (Figure 1). Based on previous reports, MODY is included in a group of genetic defects in the pancreatic β-cells limiting the ability of pancreas to produce insulin required for glucose utilization (4, 5). These patients exhibit mild or no diabetic symptoms, and their elevated glucose level can be detected only during routine blood tests. MODY has numerous subtypes depending upon the gene involved and the clinical phenotypes. Previously, 13 MODY subtypes were identified and now 14th subtype has been recently added to the list (Table 1) (6, 7).

**Table 1:** Genes associated with various maturity-onset diabetes of the young (MODY) types including frequencies and year of recognition of various MODY types.

MODY type	Gene	Chromosomal locus	Frequency (%)	Year of recognition	Reference
MODY1	HNF4α	20q13	5	1991	(8, 9)
MODY2	GCK	7p13	15–25	1993	(10)
MODY3	HNF1α	12q24	30–50	1996	(11, 12).
MODY4	PDX/IPF1	13q12.2	<1	1997	(13)
MODY5	HNF-1β	17q12	5	1997	(14, 15)
MODY6	NEUROD1	2q31	<1	1999	(16)
MODY7	KLF11	2p25	<1	2005	(17)
MODY8	CEL	9q34	<1	2006	(18)
MODY9	PAX4	7q32	<1	2007	(19)
MODY10	INS	11p15	<1	2008	(20, 21)
MODY11	BLK	8p23.1	<1	2009	(22)
MODY12	ABCC8	11p15	<1	2012	(23)
MODY13	KCNJ11	11p15.1	<1	2012	(24)
MODY14	APPL1	3p14.3	<1	2015	(7)

## History

The symptomless diabetes was reported in the non-obese children and teenagers in 1958 whose glucose level was maintained by sulfonyl-urea treatment (25). The term “maturity-onset type diabetes of Young or of the children” was given by Fajan in 1964 at the Fifth Congress of the International Diabetes Federation in Toronto to highlight its firm hereditary basis. During this period, MODY was named as type 2 diabetes, which could be managed by diet, insulin, and oral drugs (26). In 1973, it was reported that the carbohydrate intolerance in 45 patients below the age group of 25 years never reached extreme conditions, although they were treated with sulfonylureas for 16 years; however, 43 of these patients were having a strong diabetic history. A “mild” form of diabetes was reported in three families having inherited autosomal dominant and early-onset diabetes treated with sulfonylureas (27). The acronym “Maturity-onset Diabetes of the Young” (MODY) was jointly used by Tattersall and Fajans and defined MODY as “Fasting hyperglycemia” (28). The genetic studies on MODY was started on Michigan family *viz* R-W pedigree in which 74 members suffered from non-insulin dependent diabetes mellitus (29). The co-segregation of DNA polymorphism in adenosine deaminase (ADA) gene was observed after analyzing 75 DNA markers (30). Bowden et al. (31) confirmed that MODY is linked to D20S16. The monogenetic nature of MODY diabetes was confirmed after the identification of mutation in glucokinase (GCK) gene (32). The non-sense gene mutation (P.Q268X) in hepatocyte nuclear factor 4 alpha (HNF4α) gene was reported as the cause of MODY5 (11). At present, 14 MODY types specified by mutation in respective 14 genes with their etiologies are known.

## Prevalence of MODY

Maturity-onset diabetes of the young is not the prevalent diabetic form in the world population and is usually misclassified with type 1 and type 2 diabetes (33, 34). In UK, 80% of MODY patients are misdiagnosed as type 1 or type 2 diabetes (35). In developed countries, the reported frequency of MODY is 1–2% (36). Amed et al. (37) reported the minimum incidence rate of MODY in Canadian population that is 0.4 cases per 100,000 children and youth of <18 years age. However, it has been reported to be highly prevalent in certain communities including Pima Indians (38), Nauru population (39), and southern India (40). Mohan et al. (41) reported a high prevalence of MODY in Asian Indian patients. According, to Pihoker et al. (42), the minimum prevalence of MODY increases to 2.1/100,000 individuals of <20 years of age.

Till now, 14 subtypes of MODY with distinct genetic etiologies have been identified. The relative prevalence of MODY subtypes vary greatly with MODY2 and MODY3 being the most prevalent (Table 2). The relative frequency of each MODY type varies due to different recruitment data and ethnic variability.

**Table 2:** Prevalence of MODY2 and MODY3 in different countries.

Country	Prevalence of MODY2 (%)	Prevalence of MODY3	Reference
China	1	9%	(43)
Czech	31	11.5%	(44)
Denmark	10	36%	(45)
France	46–56	<25%	(10, 46, 47).
Germany	8	22.5%	(48, 49)
India	<1	9%	(50).
Italy	41–46	14%	(51–54).
North America	–	17%	(55)
Norway	–	52% clinical; 20% suspected	(56)
Spain	25–41	35%	(57, 58)
Sweden	3.5	5.2%	(59)
UK	11–20	73%	(60, 61)

## MODY Subtypes

As per Vaxillaire and Froguel (62), MODY represents a combination of genetic, metabolic, and clinical heterogenicity. MODY has several subtypes (Table 1) depending upon the involvement of genes and their mutations (deletion, splice-site, non-sense, etc.) with MODY2 being associated with enzyme GCK, while rest being associated with transcription factors.

### HNF4α-MODY (MODY1)

Hepatic nuclear factor 4 alpha (HNF4α) is an important gene located on chromosome 20 and plays a significant role in regulating the expression of liver and pancreatic β-cells (63, 64). Mutation in exon 4 (Thr^130^ → Ile) and exon 7 (Gly^268^ → Thr) of HNF4α gene is responsible for causing MODY1 (8, 9). HNF4α regulates the proteins essential for glucose transport metabolism as well as lipoprotein biosynthesis in liver cells (65–67). HNF4α mutation causes decline in insulin production except during neonatal period, where it results in hyperinsulinemic hypoglycemia thereby exhibit phenotype similar to MODY3 (8, 9, 68). MODY1 has the penetrance of less than 5% where most carriers exhibit diabetic signs before 25 years of age and about 15% patients show diazoxide-responsive hyperinsulinemic hypoglycemia history (69, 70). HNF4α mutation also leads number of pancreatic disorders like decline in high-density lipoprotein (HDL), triglyceride levels and increment in low density lipoprotein (LDL), respectively.

### GCK-MODY (MODY2)

Glucokinase enzyme also named as hexokinase D belongs to hexokinase gene family which acts as glucose sensor in pancreatic β-cells (71, 72) thus, playing critical role in glucose homeostasis. This enzyme is constitutively expressed and catalyzes the initial rate limiting step in the glycolytic pathway by ATP dependent phosphorylation of glucose to glucose-6-phosphate (71) in presence of Mg ions. GCK gene mutations upregulating the insulin secretion and resetting the glucose threshold results in enhanced fasting glucose level (73). Ellard et al. (74) reported GCK mutations in 5–6% of women who were suffering from gestational diabetes. The frequent heterozygous inactivating GCK mutations are responsible for the second most common type of MODY termed as MODY2 (75). MODY2 is characterized by mild persistent fasting hyperglycemia and low glucose-stimulated insulin secretion (75). So for as the clinical complications of GCK mutations are concerned, activating and heterozygous mutations results in persistent hyperinsulinemic hypoglycemia of infants (PHIH), while as homozygous mutations in both the alleles can result in permanent neonatal diabetes mellitus (PNDM) and mutations in single allele can result in GCK-MODY (76–78). The corresponding amino acids are very much important for GCK activity as per the reports of Galán et al. (79) who demonstrated the occurrence of two new GCK mutations Leu^165^ → Phe, Glu^265^ → lys and missense Thr^206^ → Met in Spanish families. However, some mutations do not have any visible effect on the GCK activity like Val^62^ → Met (80). Previously more than 600 GCK mutations have been reported in 10 exons of GCK gene (81).

### HNF1α-MODY (MODY3)

The hepatocyte nuclear factor 1 alpha (HNF1α) gene plays an important role in regulating expression of insulin gene in the mature β-cell and glucose transporter GLUT2 gene (82, 83). Fajans (12) revealed the association of HNF1α gene mutation with MODY 3 diabetes by conducting linkage analysis of R-W pedigree. Hyperglycemia due to HNF1α mutations can be controlled with sulfonylureas for a number of years (84). Anuradha et al. (85) studied the gene polymorphism of HNF1α and showed the high prevalence of amino acid polymorphism at codon (Ala^98^ → Val) which is associated with MODY3 diabetes. Earlier, it was demonstrated that patients with HNF1α mutations develop diabetes after the first decade, preceded by abnormal glucose-induced insulin secretion (86). MODY3 is characterized by a progressive reduction in insulin secretion. It has been shown that carriers of HNF1α mutation show increased β-cell apoptosis (87).

### PDX1/IPF1-MODY (MODY4)

Pancreatic and duodenal homeobox 1 (PDX1)/insulin promoter factor 1 (IPF1) plays significant role in regulating transcription of genes encoding: glucagon, Insulin, GLUT2, and GCK enzymes (88). IPF1 acts as key switch in maintaining the hormonal and enzymatic functions of pancreas on binding with the TAAT element of the target gene (IPF1) promoter region (89). However, mutation of IPF1 gene located on chromosome 13 results in MODY4 (13, 90). Pancreatic anomaly termed agenesis can occur due to disrupted IPF gene activity and heterozygous IPF mutations can cause defective insulin secretion thus MODY 4 diabetes, while as, homozygous mutation resulting in permanent neonatal diabetes (PND) and pancreatic exocrine insufficiency (13, 91, 92).

### HNF-1β-MODY (MODY5)

Hepatocyte nuclear factor-1β (HNF-1β) gene functions in the various developmental processes in humans like nephron and embryonic pancreas development. Mutation in HNF-1β gene results in MODY type 5 and the patients commonly exhibit notable histological anomalies showing meganephrons, and renal cysts referred as renal cysts and diabetes syndrome (RCAD) (48, 93). Additional complications associated with MODY5 are vaginal aplasia, rudimentary uterus (48), hyperuricemia, gout (94), and reduced birth weight to 900 g (15). Heterozygous Pro^159^ → Leu mutation in HNF1β gene results in impaired glucose metabolism (95).

### NEUROD1-MODY (MODY6)

Neurogenic differentiation-1 gene (NEUROD1) transcription factor is significant role in pancreatic and neuronal development and is functionally expressed in both pancreatic and neurons cells. However, its impairment results in autosomal dominant inheritance mutations similar to type 2 (16, 96, 97). Modified activity of NEUROD1 gene in carriers of Arg^111^ → Leu and insertion of cytosine residue in a polyC tract in codon 206 (206 + C) of exon2 were largely responsible for development of diabetes (16). Mutation in basic-loop-helix transcription factor NEUROD1 is responsible for MODY6 (75).

### KLF11-MODY (MODY7)

Krueppel-like factor 11 (KLF11) gene is located on chromosome 2 and plays important role in pancreatic β-cell functioning (98, 99). KLF11 mutation affects the pancreatic β-cell function by modulating the expression of certain free radical scavengers such as catalase and superoxide dismutase (SOD). The expression level of these antioxidant enzymes is reported to be low in pancreatic islets; however, the over expression of these enzymes protects β-cells against glucolipotoxicity (100). The occurrence of several uncommon variants Ala^347^ → ser, Thr^220^ → Met, and Gln^62^ → Arg of KLF11 gene associated with familial diabetes which severely disrupts transcriptional machinery was analyzed by genetic analysis and mutation in KLF11 results in MODY termed MODY7 (17).

### CEL-MODY (MODY8)

The main ingredient of pancreatic juice is carboxyl-ester lipase (CEL) encoded by carboxyl-ester lipase gene (9q) and plays an important role in newborns by digesting milk and hydrolyzing dietary esters in duodenum (101, 102). A single base deletion resulted in the altered C-terminal sequencing, thus causing the monogenetic disease, i.e., MODY8. On the other hand, a single base insertion (1686delT; C563fsx673) causes polygenic diabetes resulted by truncation of the CEL (103), thus suggesting a frame shift deletion mutation in variable number tandom repeats (VNTR) in the 11th exon of CEL gene. In addition, common as well as rare CEL mutations also affect exocrine and endocrine functioning of pancreas leading to MODY diabetic syndrome (MODY8).

### PAX4-MODY (MODY9)

Paired box gene 4 (PAX4) is a transcription factor which is a member of PAX family located on chromosome 7 and regulates fetal development, cancer growth, commitment of progenitor cells to various islet cells and also represses the promoter activity of insulin and glucagon (104, 105). PAX4 is required for the regeneration of β-cells in adults and its mutation blocks or inhibits β-cell proliferation (106). Two PAX4 gene mutations *viz* Arg^164^ → trp and IVS7-IG>A were reported as the cause of monogenetic form of diabetes in Thai population termed MODY 9 (19).

### INS-MODY (MODY10)

The insulin gene (INS) which is located on chromosome 11p15.5 encodes the proinsulin precursor of insulin, its mutation cause defect in NF-κB (nuclear factor kappa-light-chain-enhancer of activated B cells) and thus leads to MODY10 (107, 108). The mutations in cysteine residues (example Cys^96^ → tyr) leads to disruption of disulfide bonds, other mutations like Ala^24^ → Asp, phe^48^ → Cys, Arg^89^ → Cys, His^29^ → Asp, Leu^35^ → Pro, Gly^84^ → Arg, Cys^96^ → Ser, S^101^ → Cys, and Tyr^103^ → Cys have been observed in young diabetic patients (21).

### BLK-MODY (MODY11)

Lymphocyte kinase tyrosine kinase (BLK-B) encoding the receptor protein tyrosine kinase, plays a significant role in thymopoiesis in immature T cells (109). Drebin et al. (110) cloned human homolog of murine BLK and concluded that like murine BLK gene human BLK is expressed only in β-cells. The obese feature of diabetes was reported to be linked with chromosome 8p23 gene locus (111). BLK expression is altered in response to this Ala^71^ → Thr substitution (22). The BLK gene mutation essentially affects MIN6 B-cells (a highly differentiated B-cell line) and is responsible for MODY11 (22).

### ABCC8-MODY (MODY12)

Before 40 years of age, the ABCC8 mutations were reported in the cohort of adult type 2 diabetic patients (112). The insulin secretion which regulates blood sugar levels is mediated by the ATP-binding cassette transporter subfamily C member 8 (ABCC8) encoding sulfonyl-urea receptor 1 ATP-binding cassette transporter subfamily C member 8 (ABCC8), and encodes sulfonyl-urea receptor 1 (SUR1) which is the subunit of K-ATP channel (ATP-sensitive potassium channel) found across β-cell membrane. ABCC8 gene mutation results in sulfonylureas responsive MODY termed MODY12 and clinical anomalies can occur both due to activating and inactivating mutations of ABCC8 gene. Congenital hyperinsulinism occurs as a result of dominantly inherited inactivating mutations, while the permanent or transient neonatal diabetes (TNDM OR PNDM) is caused due to activating mutations or recessive loss-of-function mutations (113). Four unique mutations (Glu^100^ → Lys, Gly^214^ → Arg, Gln^985^ → Arg, and Asn^125^ → Asp) in ABBC8 gene was found in susceptible MODY patients (23), the heterozygous ABCC8 mutations were reported in 7/85 (8%) non-obese sulfonylureas sensitive patients with BMI < 30 kg/m^2^ with clinical features similar to HNF1α/4α MODY (23).

### KCNJ11-MODY (MODY13)

The potassium channel, rectifying subfamily J, having member 11 (KCNJ11) which encodes human BIR (beta cell inward rectifier or Kir6.20) that is intron less coding gene was determined (114). Earlier, it was predicted that KCNJ11 gene mutations might result in neonatal diabetes. The sequence analysis revealed the occurrence of six new heterozygous missense mutations in 10 out of 29 patients and among these four patients exhibit Arg^201^ → His mutation concluding that neonatal diabetes is caused by heterozygous mutations of the KCNJ11 gene (115). The onset and severity of diabetes occurring due to the missense KCNJ11 gene mutation is variable and spans up to three generations (116). Massa et al. (117) revealed the occurrence of five different heterozygous mutations including two novel mutations by screening the KCNJ11 gene in eight Italian patients and concluded that KCNJ11 gene mutations are the common cause of PNDM. The KCNJ11 gene mutation due to the disrupted subunit interaction results in severe conditions like channel inactivation, which was found to be associated with Arg^301^ mutation that determinately cause hyperinsulinism (118).

### APPL1-MODY (MODY14)

The knockdown mutation of APPL1 gene results in apoptosis and the overexpression resulted in dysmorphic phenotypes and delay in development, as was revealed from the previous reports of APPL1 gene expression in Zebra fish (119). In adapter protein, phosphotyrosine interaction domain and leucine zipper containing protein-1 (APPL1), both missense and non-sense heterozygous loss of function results in asp-to-asn (Asp^94^ → Asn) substitution, while non-sense mutation results in Leu^552^ → X (Leu^552^ → X) substitution (7). The mutation in APPL1 gene was reported by Prundate et al. (7) as a cause of MODY14.

## Diagnosis/Clinical Features and Management

Some of the major diagnostic characteristics of MODY given by Vaxillaire and Froguel (62) are as follows:

- Elevated glucose level (hyperglycemia) is diagnosed in early age (before 25 years) in one or two suspects of the diabetic family.
- Autosomal dominant inheritance is showing vertical transmission through at least three generations. Similar phenotype shared by diabetic family members.
- Insulin is not required by patients up to 5 years after initial diagnosis. As far as C-peptide level is concerned it tends to remain low even in patients on insulin therapy.
- Functional impairment in the pancreatic β cells.
- Does not show the normal diabetic features like obesity, etc.

**Figure 2:**
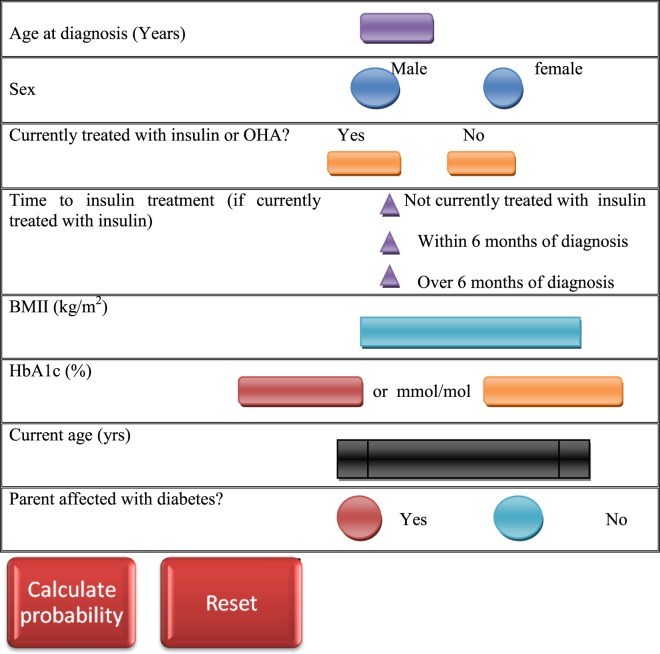
Maturity-onset diabetes of the young (MODY) testing calculator (120). The MODY probability is calculated by entering clinical features of the patient in the probability calculator.

Besides the clinical features, probability of MODY occurrence can be calculated by using standardized MODY probability calculator (120) (Figure 2), and the clinical features may vary with the MODY type in consideration. However, clinical diagnostic characteristics of the most common MODY types are as follows.

### GCK-MODY2

1. The fasting elevated glucose level observed is ≥5.5 mmol/l (98%) which is stable (from months up to years) and persistent (for at least three independent occurrences).
2. It has been observed that the Hb1Ac is typically just above the normal level and in some cases rarely exceeds 7.5% (3).
3. As per the reports of Stride and Hattersley (3), there had been a little (in European population 71% of the patients had <3 mmol/l) increment in an Oral Glucose Tolerance Test (OGTT) [(2 h glucose) − (fasting glucose)].
4. Parents of the affected individuals may have mild type 2 diabetes with lesser complications or may not be diabetic at all. However, further testing revealed that one parent will commonly have a moderately raised fasting hyperglycemia (5.5–8 mmol/l) except in the subjects where the mutation has originated *de novo* (3).

### HNF1α-MODY3

1. Lower insulinogenic index and lower early insulin response is observed in HNF1α mutants compared with the familial non-mutants even when blood glucose level is normal (86).
2. Patients are typically lean and without characteristic insulin resistance (121).
3. Positive β-auto-antibodies (IA-2) were reported in 25% (7/28) of HNF1α patients, and these patients develop diabetes much later than those with negative autoantibody. It was further reported that in such patient’s glycemia was difficult to control (122).
4. C-reactive protein level tends to be lower in MODY3 patients (123).
5. Glycosuria often observed in patients with low renal threshold due to diminished expression of high affinity low-capacity co-transporter 2 (SGLT2) which decreases the glucose reabsorption from kidney renal tubules (124).

For the genetic diagnosis of MODY, only those subjects are selected which fit into the standard clinical criteria of disease (125). For testing the monogenetic diabetes, DNA is isolated from the patient’s blood sample which is then analyzed for mutation(s). Next-generation sequencing (NGS) strategies according to Ellard et al., (126), Johanson et al. (127) can be employed to identify MODY gene mutation. By direct sequencing, MODY can be diagnosed approximately with 100% sensitivity (128). According to Nyunt et al. (129) suspected cases when not diagnosed by routine screening technique can be detected by multiple ligase dependent probe amplification (MLPA) technique. There are some biomarkers that effectively differentiate MODY from other forms of diabetes and are thus effectively used for accurate testing. MicroRNAs possibly can be used as biomarkers of HNF1α-MODY, as it has been observed that the inducible expression of Pro^291^ → fsinsC-HNF1A in INS-1 cells resulted in a significant upregulation of three miRNAs (miR-292-3p, miR-103, and miR-224) (130). Furthermore, the elevated expression levels of miR-103 and miR-224 could be detected in the serum of HNF1α-MODY carriers compared with MODY-negative family controls. The high HDL cholesterol level in MODY3 patients acts as the biomarker for differentiating MODY from type 2 diabetes (131). Sanger sequencing is the most reliable method for MODY diagnosis (70). A correct molecular diagnosis helps in the optimal treatment of disease. As β-cell function deteriorates over time, pharmacologic therapy becomes necessary to prevent diabetes-related complications. Once the gene mutation is accurately detected, only then the first degree relatives with MODY symptoms can be accurately diagnosed with the disease (132).

Maturity-onset diabetes of the young patients can be managed by diet alone for many years and sulfonylureas is also recommended to be very effective for managing glucose level for more than 30 years (12). Patients diagnosed with GCK mutations usually need no treatment, but require medication in case of pregnancy and gestational diabetes mellitus (GDM). HNF1α and HNF4α MODY patients are particularly sensitive to sulfonylureas, but in the later stages should be supplemented with the addition of insulin (133). In HNF1α MODY patients (134) reported higher mean A1C levels in those taking insulin (7.5%) vs. oral agents (6.7%). Patients with HNF1α and HNF4α mutations are typically sensitive to sulfonylureas. Low-dose sulfonylureas (20–40 mg gliclazide regularly) can aid in sustaining the MODY patients for decades (135). Drugs like nateglinide and liraglutide lower the postprandial glucose levels in HNF1α MODY subjects (136, 137). The pathophysiology associated with MODY gene mutations and various possible treatments for different MODY types are shown in (Table 3).

**Table 3:** Maturity-onset diabetes of the young (MODY) gene functions and pathophysiology and possible treatment.

MODY gene	Gene function	Pathophysiology	Treatment
HNF4α	Transcription factor (nuclear factor)	β-Cell dysfunction, neonatal hyperinsulinemia	Sulfonylureas
GCK	Hexokinase (catalyze the initial step in the glycolytic pathway)	β-Cell dysfunction, fasting hyperglycemia from newborn	Diet
HNF1α	Transcription factor (homeodomain), regulating insulin gene transcription	β-Cell dysfunction, glycosuria	Sensitive to sulfonylureas
PDX/IPF	Transcription factor (homeodomain)	β-Cell dysfunction and pancreatic agenesis	Diet or AD insulin
HNF-1β	Transcription factor (homeodomain)	β-Cell dysfunction and renal anomalies, genital anomalies. Pancreatic hypoplasia	Insulin
NEUROD1	Transcription factor (BHLH)	β-Cell dysfunction, adult onset diabetes	OAD or insulin
KLF11	TGF-β	β-Cell dysfunction, similar to type 2 diabetes mellitus	OAD or insulin
CEL	Controls exocrine and endocrine functions of pancreas, pathogenesis of pancreatic malabsorption and diabetes mellitus	Pancreas exocrine, endocrine dysfunction and pancreas	Insulin or OAD or diet
PAXA4	Transcription factor, play role during cancer growth and fetal development	β-Cell dysfunction, possible ketoacidosis	Insulin or OAD or diet
INS	Regulate beta cells of activity Langerhans cells	Insulin gene mutation, PNDM	Diet or OAD, insulin
BLK	B-cell specific, tyrosine kinase functions in signal transduction	Insulin secretion defect, overweight	Insulin or diet (OAD)
ABCC8	Regulate insulin secretion by linking cellular metabolism to electrical activity of plasma membrane	ATP-sensitive potassium channel dysfunction, PND TND	OAD (insulin)
KCNJ11	Regulate glucose-induced insulin secretion in pancreatic cells	Homozygote: neonatal diabetes	Insulin OAD or diet
APPL1	Involved in signal transduction	Dysmorphic phenotypes, development delay	Diet or OAD, insulin

## Genetic Testing Methodologies

Different genetic testing methodologies like linkage analysis and direct sequencing were used by different workers for investigating gene mutations associated with MODY diabetes, which are summarized below (Table 4). Fajans (12) by linkage analysis investigated 360 members of the diabetic family (R-W Pedigree) by taking into account MODY patients. Linkage analysis confirmed that the HNF1α gene which is located on chromosome 12q24.2 and is responsible for causing MODY3. Association of the GCK gene mutations with the early-onset diabetes has been reported with the help of linkage analysis (32). Froguel et al. (32) reported hyperglycemia in 125 out of 304 examined subjects, with 33% exhibiting clinical characteristics of maturity early-onset diabetes termed MODY2. In addition to this, they also analyzed that there was statistically significant evidence of genetic heterogeneity with an estimated 45–95% of the 16 families showing linkage to GCK. Yamagata et al. (8, 9) isolated and partially sequenced the human HNF4α gene in R-W pedigree; following genetic screening identified thr130ile mutation in exon 4 and Gly^268^ → Thr mutation in exon 7 of the HNF1α gene. Finally, confirmed the association of early-onset diabetes with HNF4α gene mutation.

**Table 4:** Genetic testing methodologies (genetic screening and linkage analysis).

Gene studied	Methodology	Experimental results	Reference
Reported 11 MODY genes	Linkage analysis	Gene causing maturity-onset diabetes of the young (MODY) is tightly linked with chromosome hepatocyte nuclear factor 4 alpha (HNF1α) (20q12-q13.1)	(12)
GCK	Linkage analysis	GCK mutations a cause of MODY2.	(32)
HNF4α	Sequencing and screening	Verified gln268-to-ter mutation in HNF4A encoding gene	(8, 9)
IPF1	Linkage analysis	Pancreatic agenesis, pancreatic insufficiency and permanent neonatal diabetes (PND) due to IPF mutation	(13)
NEUROD1	Sequencing, gel shift assay, radioimmunoassay cell culture, and transfections.	Disrupted activity of NEUROD1 gene in carriers of R111L and 206 + C mutations was largely responsible for development of type 2 diabetes	(16)
KLF11	EMSA, random oligonucleotide binding, EMSA, luciferase reporter, and chromatin immunoprecipitation	Rare (Ala347Ser and Thr220Met) variant with familial young type 2 diabetes and Gln62Arg variant was found to be linked with type 2 diabetes	(17)
HNF1β	Gene sequencing	10 novel mutations in HNF1β gene with 26% families having MODY and 39% with renal cysts and diabetes syndrome	(15)
CEL	Linkage, DNA sequencing	Single base pair deletion (1686delT; C563fsx673) in 11th exon of CEL gene	(103)
INS	PCR-Single strand sequencing	Observed 6 novel mutations: H29D, L35P, G84R, C96S, S101C, and Y103C	(21)
PAX4	PCR-SSCP, restriction fragment length polymorphism	Observed two unusual mutations R164W and IVS7-IG>A in PAX4, with E164 associated with diabetes	(19)
BLK	Sequencing, RT-PCR. Florescent staining	BLK expression is altered in response to Ala71Thr substitution	(22)
ABCC8	Sequencing	4 novel mutations 4 novel mutations (E100K, G214R, Q985R, and N125D in ABCC8 gene as the cause of MODY12)	(23)
KCNJ11	WES, linkage analysis	Observed one mutation (p.Glu227Lys) in KCNJ11 as the cause of MODY13	(24)
APPL1	WES approach	In the diabetic families a missense asp94-to-asn (D94N) and non-sense leu552-to-X (L552X) APPL1 gene mutations were frequently observed	(7)

Stoffers et al. (13) on the basis of linkage analysis, mode of transmission and phenotypic variations suggest the occurrence of fourth type of early-onset diabetes termed MODY 4 caused due to mutation of IPF1 gene locus. Stoffers et al. (13) reported that pancreatic agenesis is caused by homozygous mutation in IPF, which results in exocrine pancreatic insufficiency and permanent neonatal diabetes (PND). Malecki et al. (16) by direct sequencing of DNA samples from 94 subjects with type 2 diabetes, verified that the disrupted activity of NEUROD1 gene in carriers of Arg^111^ → Leu (arginine to leucine substitution at codon 111) and insertion of cytosine residue in a polyC tract in codon 206 (206 + C) in exon 2 of NEUROD1 were largely responsible for development of type 2 diabetes. The NEUROD1 results in autosomal dominant inheritances similar to type 2 diabetes (16). Neve et al. (17) on investigating KLF11 gene by direct sequencing and various other testing methodologies like-random nucleotide binding; chromatin immune-precipitation demonstrated that KLF11 gene by binding to the insulin promoter maintain pancreatic β-cell activity. Genetic analysis revealed the occurrence of two uncommon variants Ala^347^ → Ser and Thr^220^ → Met which are associated with familial young type 2 diabetes and severely disrupts transcriptional machinery. Moreover, in North European population while analyzing 1,696 type 2 diabetic patients and 1,776 normal individuals. Neve et al. (17) reported that polymorphic Gln^62^ → Arg variant was found to be linked with type 2 diabetes. This Gln^162^ → Arg variant was found to deviate the KLF11 co-repressors mSin3A-binding property which results in altered insulin promoter activation and hence results in reduced insulin gene expression in pancreatic β-cells along with other complications like reduced plasma insulin. Thus, it was concluded that KLF11 variants assist in developing diabetes.

Edghill et al. (15) sequenced HNF-1β gene in 160 non-associated patients suffering from renal diseases and observed HNF1β mutations in 14% (23/160) including 10 unique mutations (Val^61^ → Gly, Val^110^ → Gly, Ser^148^ → Leu, Lys^1565^ → Glu, Gln^176^ → X, Arg^276^ → Gln, Ser^281^ → fsinsC, R^295^ → P, His^324^ → fsdelCA, and Gln^470^ → X). Furthermore, out of these 23 novel mutations; diagnosed renal cysts in 19 (83%) and diabetes in 11 (48%). Only 6% (26%) of families fulfill the diagnostic characteristics for MODY, and 39% families had RCAD. Thus, by sequence analysis observed a definite association between HNF1β gene mutation and renal abnormalities. Raeder et al. (103) examined a large family with autosomal dominant diabetes typically detected before 40 years of age with primary β-cell dysfunction. Linkage analysis and DNA sequencing of CEL gene reveals that the single base deletion (1686delThy; C563fsx673) at 11th exon was found in all risk family members having both diabetes and fecal elastase deficiency. Plengvidhya et al. (19) tested 46 Thia type 2 diabetic patients of <35 years of age by PCR-single-stranded conformation polymorphism (PCR-SSCP), this was succeeded by direct screening of PAX4 mutations; final observation of variants was performed by restriction fragment length polymorphism. Two unusual mutations Arg^164^ → Trp and IVS7-IG > A of PAX4 gene were identified. R164W mutation was shown to be associated with diabetes, by affecting the function of PAX4 gene (suppressing the insulin and glucagon promoter action). Edghill et al. (21) in European population observed INS mutation in 33 of 141 probands (diagnosed at < 6 months age), 2 of 86 (diagnosed between 6 and 12 months age) and none of 58 (diagnosed between 12 and 24 years of age) by PCR-single strand sequencing. Edghill et al. (21) observed that 46% of the complications were due to the three already described mutations *viz* Arg^24^ → Asp, Phe^48^ → Cys, and Arg^89^ → Cys and also identified six unusual mutations: His^29^ → Asp, Leu^35^ → Pro, Gly^84^ → Arg, Cys^96^ → Ser, Ser^101^ → Cys, and Tyr^103^ → Cys. Borowiec et al. (22) by using the techniques of immune florescence staining, western blotting, sequencing, RT-PCR, examined 6 MODY families and identified 410 sequence variations in 732 kb of genomic sequence at 8p23. Based on the results observed—five mutations with the frequency of <1% comprises four non-coding mutations and Ala^71^ → Thr substitution, they summarized that BLK expression is altered in response to this Ala^71^ → Thr substitution.

Bowman et al. (23) sequenced ABCC8 gene in 85 patients (BMI < 30 kg/m^2^) with no familial diabetic history and observed ABBC8 mutations in 7 (8%) probands. In addition, to four unique mutations (Glu^100^ → Lys, G^2ly14^ → Arg, Gln^985^ → Arg, and Asn^125^ → Asp), some previously reported heterozygous mutations were also reported in four patients. Final results showed that only four probands fulfill the clinical criteria of early-onset diabetes termed MODY 12 which was having clinical pathologies identical to that of HNF1α MODY and can be diagnosed before 25 years of age. Bonnefond et al. (24) performed WES (Agilent-sureselect capture/Illumina Golden Gate assay) and linkage analysis in three affected and one unaffected subjects in MODY-X family, and observed 69 mutations with only one mutation (p.Glu^227^ → Lys) in KCNJ11 gene associated with diabetes (LOD-score of 3.68). Thus, confirming KCNJ11 gene mutation as the cause of early-onset diabetes termed MODY13. Prundate et al. (7) studied loss-of-function mutations in APPL1 gene in 60 families (52 from US and 8 from Italy) by following WES approach. However, in Italian families, normal subjects of >55 age were also included in the study. Both missense and non-sense heterozygous loss-of-function mutations have been reported to occur in APPL1 gene, and missense mutation results in asp-to-asn (Asp^94^ → Asn) substitution while non-sense mutation can result in Leu^552^-to-X (Leu^552^ → X) substitution.

## Conclusion and Future Perspective

Researchers have been effectively working to investigate the genetic determinants and pathophysiology of MODY by means of various powerful approaches (based on genome and cell biology). These studies have been very promising and insightful which has greatly extended our understanding of both normal and pathologic β-cell biology as well as regulation of insulin secretion in humans. At clinical level, MODY requires correct and accurate methods of diagnosis as well as to distinguish it from diabetes type 1 or type 2 to avoid the unnecessary insulin or sulfonylureas treatment, which may severely affect the patient’s health.

In future, translational biology and integrative genomic research and studies on both monogenic and polygenic forms of diabetes are required, which will not only provide novel insights but, also broaden our understanding in terms of pathophysiology, treatment and cure of diabetes. Currently, application of NGS has emerged a key factor to decipher the large amount of known genetic defects underlying pancreatic islet β-cell dysfunction in diabetic patients. As there is increased rate of MODY across the globe, NGS can serve as one of the potential and rapid molecular based diagnostic tool as well as to identify novel genetic etiologies of familial or atypical early-onset diabetes. Transcriptomic and secretomic analysis related to MODY may provide a significant molecular insight into the disease and its procurement. Besides genomic approaches, stem cell technology can also be another novel and effective disease model to study and therapy for the diabetes. Use of pluripotent stem cells (PSCs) can help us to better understand the molecular mechanisms underlying different forms of diabetes (MODY). In addition, metabolomic profiling can also be one of the important approaches to study and differentiate MODY with other diabetes.

## Author Contributions

PF and BG conceived and designed this review. PF, KN, US, and TH collected the literature for this review. PF, KN, and SA wrote the manuscript draft. BG, SA, and SM edited this manuscript. All the authors gave final shape to this manuscript.

## Conflict of Interest Statement

The authors declare that the research was conducted in the absence of any commercial or financial relationships that could be construed as a potential conflict of interest. The reviewer LB and handling Editor declared their shared affiliation.
